# Information, Transparency, and Equity: Disaggregated Data by Race/Color as a Tool for Public Health Management

**DOI:** 10.1590/0037-8682-0305-2025

**Published:** 2025-12-19

**Authors:** Marcos Vinicius da Silva Cordeiro, Rita de Cássia Duarte Lima, Guilherme Loureiro Werneck, Ethel Leonor Noia Maciel

**Affiliations:** 1 Universidade Federal do Espírito Santo, Programa de Pós-Graduação em Saúde Coletiva, Vitória, ES, Brasil.; 2 Ministério da Saúde, Secretaria de Vigilância em Saúde e Ambiente, Brasília, DF, Brasil.; 3 Universidade do Estado do Rio de Janeiro, Instituto de Medicina Social, Rio de Janeiro, RJ, Brasil.

The National Health Surveillance Policy defines health surveillance as a continuous and systematic process of collecting, consolidating, analyzing, and disseminating information on health. In this context, health surveillance is responsible for gathering information and providing guidance for managing the health status of populations and territories^1^.

The disaggregation of health data by race/color is an important requirement for the planning, implementation, and evaluation of public health policies. In Brazil, this mechanism enables the identification of illnesses, service access, and health outcomes, reflecting the social determinants that sustain these inequalities.

In Brazil’s Unified Health System (UHS), institutional racism is acknowledged as a determinant of health, as established by the National Policy for the Comprehensive Health of the Black Population^2^, which includes people who self-identify as Black and Brown/mixed race and defines the disaggregation of data by race/color as a strategic element for the health of the Black population. Furthermore, the systematic collection, analysis, and dissemination of these data are mandatory.

However, a gap exists between the publication of the ordinance and its effective implementation. In March 2023, the Secretariat of Information and Digital Health presented a set of measures intended to qualify the race/color field^4^ in national health information systems to the Tripartite Inter-Manager Commission in compliance with Ordinance No. 344/2017^3^.

Among these actions, the following actions stand out: restricting the use of code 99 (no information) in Brazil’s national health registry, namely CadSUS, with a requirement to update this field in legacy records; cross-referencing of data with the data in Primary Care Health Information System (SISAB) databases for automatic requalification; and technical adjustments in the Cancer Information System (SISCAN), Outpatient Information System (SIA), and Hospital Information System (SIH) to block new records without race/color information, effective as of April 2023. These operational changes aimed to improve the completeness of the race/color variable and strengthen the UHS analytical and managerial capacity. Despite these advances, underreporting and incomplete race/color data remain major challenges. Variability across systems highlights the need for federative agreements to ensure mandatory completion and link indicators to goals, making technical measures effective in implementing equity policies.

The publication of the Epidemiological Bulletin on the Health of the Black Population in 2023 represents a structural response to the gap described earlier. The first volume^5^ presents data on vital statistics, maternal and child mortality, leading causes of death by race/color, vaccination data for the Black population, and the incidence and mortality associated with sickle cell disease. The data show that White women have proportionally greater access to the recommended number of prenatal consultations than Black and Indigenous women. The proportion of live births with low birth weight increased among Black children in 2020 across all weight brackets (<2,500 g, <1,500 g, <1,000 g). Additionally, Black women accounted for over 65% of maternal deaths in the same year.

Regarding vaccination, the lack of information on race/color is particularly notable; approximately 92.7% of vaccine dose records for 14 vaccines in the national childhood schedule did not include this variable, thus hindering targeted strategic planning for vaccinating the Black population.

Another relevant point is the newly released information on sickle cell disease in Brazil presented in this volume, which highlights the challenges in obtaining data on this condition, leading to its inclusion in the list of notifiable diseases to improve surveillance and monitoring efforts^6^.

The second volume^7^ of the bulletin presents data on infectious diseases such as Human Immunodeficiency Virus/Acquired Immunodeficiency Syndrome (HIV/AIDS), syphilis, viral hepatitis, tuberculosis, malaria, and cutaneous leishmaniasis. The prevalence of AIDS among Black people exceeded 60% in 2021. In the analysis of AIDS-related deaths from 2011 to 2021, there was an increase of approximately eight percentage points among Black individuals, reaching over 60% of deaths in the final year. The incidence of syphilis in pregnant White women was decreased. In contrast, among Black women, the incidence increased by three percentage points in the same period. Among congenital syphilis cases throughout the analyzed period, more than 70% involved Black women, which aligns with prenatal care data, revealing that most of these women did not complete the seven consultations recommended by the World Health Organization, posing a challenge to the elimination of the vertical transmission of syphilis in the country.

Similarly, the analysis of tuberculosis cases among the Black population has exposed ongoing disparities. In 2021, of the 5,072 tuberculosis-related deaths recorded in Brazil, 3,267 cases involved Black individuals (64.4%). This is a significant indicator for management as it concerns a curable disease with treatment available free of charge through the UHS. It is essential that these data are used to guide specific strategies that will contribute to the elimination of tuberculosis by 2030.

It is important to highlight that methodologically, the bulletins utilized morbidity and proportional mortality data, as it was not possible to estimate risk at the time, because the 2022 Demographic Census databases with race/color breakdowns were not available. Nonetheless, the proportional data revealed an unequal distribution of disease burden and mortality outcomes between Black and White populations, considering that the Black population accounted for approximately 55% of Brazil’s population in 2022, while the proportional mortality rates for AIDS and tuberculosis among Black individuals exceeded 60%.

To ensure transparence and agility in the dissemination of data disaggregated by race/color, the Secretariat of Health Surveillance and Environment (SHSE) partnered with the National Center for Epidemiological Intelligence (NCEI) and launched the “Epidemiology and Inequalities” dashboard (https://www.gov.br/saude/pt-br/composicao/svsa/cnie) in early 2025. The platform compiles epidemiological data disaggregated by race/color for major diseases affecting the Black population, such as tuberculosis, hepatitis B and C, acquired syphilis, syphilis in pregnant women, congenital syphilis, self-inflicted violence, and interpersonal violence. This tool serves as an important support instrument for local and state management.

One of the challenges in producing qualified information in health information systems is the incompleteness of race/color variables. In many health conditions, the completion of this information requires greater attention from the management at all levels of government. Additionally, enhancing communication with healthcare professionals and the public regarding the relevance of race/colour variables is necessary for taking actions to combat racism in Brazil. To fill this gap, the SHSE, through the General Coordination of Epidemiology in Services (GCEP) and the Health Services Epidemiology Strengthening Program (HSESP), launched the Course on Analysis of Health Indicators of the Black Population in 2025. The course is aimed at health-surveillance service workers, students and professors in the health field, UHS users, counselors, and representatives of social movements. Its objective is to promote awareness about the sociocultural processes of racism and their impact on the health of the Black population, as well as their influence on the analysis and construction of epidemiological indicators (https://campus.paho.org/pt-br/curso/curso-de-analise-de-indicadores-de-saude-da-populacao-negra-brasil-20240).

Therefore, the available management tools still require greater dissemination and integration with educational strategies by health professionals, academia, and civil society. It is necessary to establish a collective commitment to addressing the social inequalities that affect the structure and function of the UHS. Similarly, it is urgent to encourage epidemiological research that not only highlights differences in health conditions between Black and White populations but also examines the distribution of disease burden in connection with structural and institutional racism.
